# Conversion of Amazonian Biomass (Tucumã Bark) into Porous Carbon Electrodes for Energy Storage

**DOI:** 10.1021/acsomega.5c08826

**Published:** 2025-12-11

**Authors:** L. L. A. Teixeira, V. V. Oliveira, J. C. M. da Costa, R. R. Passos, L. A. Pocrifka

**Affiliations:** † Chemistry Department, Laboratory of Electrochemistry and Energy, 67892 Federal University of Amazonas, Manaus 69077-000, Amazonas, Brazil; ‡ 37871 Federal University of Pará, Belém 66075-900, Pará, Brazil

## Abstract

The valorization of biomass is a key strategy for producing high-value materials aimed at sustainability. In this study, waste from tucumã (*Astrocaryum aculeatum*), a native fruit of the Amazon region, was investigated as a precursor for the fabrication of porous carbon electrodes intended for energy storage applications. The synthesis process involved the initial pyrolysis of the biomass, followed by chemical activation using potassium hydroxide (KOH) at varying biochar/KOH ratios (1:1, 1:3, and 1:5). The resulting materials were characterized by scanning electron microscopy (SEM), X-ray diffraction (XRD), Raman spectroscopy, and nitrogen adsorption–desorption analysis. Electrochemical performance was evaluated using cyclic voltammetry (CV), galvanostatic charge–discharge (GCD), and electrochemical impedance spectroscopy in a 1 M KOH electrolyte within a potential window of −1.0 to 0.0 V. All samples exhibited characteristic (002) and (101) planes in the XRD patterns and D and G bands in the Raman spectra. The specific surface areas were 636, 1932, and 2468 m^2^ g^–1^, while the specific capacitances, calculated from GCD measurements, were 84, 146, and 298 F g^–1^, respectively. The electrode with the highest capacitance also demonstrated excellent cycling stability, retaining 98% of its initial capacitance after 7000 cycles at a current density of 7 A g^–1^ in KOH electrolyte. These results highlight that increasing the KOH dosage during activation is a critical factor in achieving carbon electrodes with optimized structural and electrochemical properties, emphasizing the potential of Amazonian biomass waste as a sustainable raw material for high-performance supercapacitor development.

## Graphical abstract

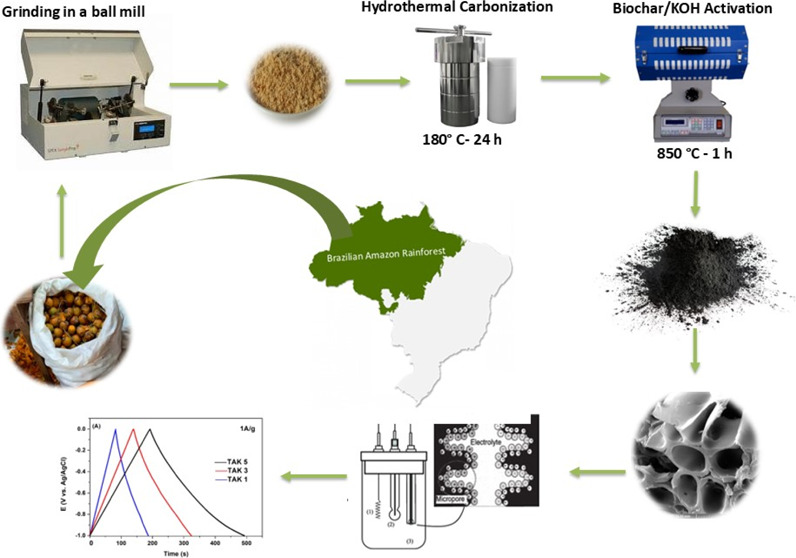

## 1 Introduction

The bioeconomy has increasingly emphasized the use of technological advances to develop new materials and services in a sustainable manner. This broad field encompasses the economic utilization of biomass and biological processes, integrating biodiversity with innovation.^1^ In this context, the Amazon region-one of the most biodiverse areas on the planet-offers tremendous potential as a source of carbonaceous residues that can be transformed into high-value materials such as activated carbon.^2^ Such materials exhibit a wide range of functionalities, including their use as high-performance electrodes in next-generation supercapacitors for energy storage.

Supercapacitors (SCs) store energy through both physical and electrochemical processes. In particular, electric double-layer capacitors (EDLCs) operate primarily via electrostatic mechanisms, where energy is stored by ion adsorption at the electrode/electrolyte interface, forming an electric double layer.^3^ SCs exhibit high power density and can deliver or absorb energy rapidly, making them suitable for applications that demand fast energy uptake and release, such as electric vehicles, renewable energy buffering systems, and portable electronics.^4^ Moreover, they can withstand millions of charge–discharge cycles, ensuring long operational lifetimes and excellent energy efficiency.^5^

EDLC-type SCs require electrode materials with a high surface area to maximize charge storage. Carbon-based materials-such as nanotubes, graphene, and activated carbon as well as certain metal oxides are frequently investigated for this purpose.^6,7^ Among these, activated carbon stands out because it combines a high specific surface area, excellent electrical conductivity, tunable porosity, and low cost. In addition, it can be derived from abundant biomass residues, offering an environmentally friendly and economically attractive route for the sustainable manufacture of energy-storage devices.^8,9^

Various synthesis strategies have been explored for the production of such carbonaceous materials. A widely adopted approach involves thermal treatment of biomass (carbonization) followed by chemical activation.^10^ Carbonization, or physical activation, typically consists of pyrolysis under an inert atmosphere at temperatures ranging from 600 to 900 °C.^11^ Chemical activation, in turn, employs acidic, basic, or saline activating agents-most commonly H_3_PO_4_, KOH, and ZnCl_2_-followed by thermal treatment, and has proven especially effective in creating hierarchical porous networks that promote ion transport and charge storage.^11^

A wide variety of biomass sources has been investigated for the production of biochar intended for use in SC electrodes. For example, Lee et al.^12^ used coconut shells activated with KOH, achieving 449 F g^–1^ at 1 A g^–1^ in a 6 M LiNO_3_ aqueous electrolyte. Ajay et al.^13^ employed orange peels, obtaining a capacitance of 144 F g^–1^ at a scan rate of 2 mV s^–1^ in 1 M KOH by cyclic voltammetry (CV). Similarly, Tripathy et al.^14^ used banana peels activated in a single step with ZnCl_2_ and FeCl_3_, achieving 74 F g^–1^ and 218 F g^–1^, respectively, and demonstrating excellent performance. These studies highlight that selecting an appropriate strategy for converting biomass into activated carbon is crucial for achieving high efficiency and specific capacitance.

Despite these advances, the conversion of Amazonian residues into high-value energy-storage materials remains notably underexplored. The Brazilian Amazon, with its unmatched biodiversity and the continuous production of regional fruits, generates large volumes of agro-industrial waste that remain largely unutilized. Peels, seeds, and fibers from typical Amazonian fruits-such as açaí, buriti, and tucumã-are discarded in significant quantities, representing a strategic potential source of low-cost raw materials.^15^ Rich in carbon and endowed with favorable structural characteristics, these residues can be converted into high-performance carbonaceous materials, strengthening the regional bioeconomy and offering a sustainable alternative for the development of next-generation energy-storage devices.

Within this context, the peel of the tucumã fruit (*Astrocaryum aculeatum*) stands out as an abundant yet largely unexploited residue. This fruit, widely consumed in northern Brazil, generates tons of discarded peel annually, creating both an environmental liability and a strategic opportunity for the production of high-value materials. Utilizing this residue not only reduces raw material costs but also mitigates the environmental impacts associated with improper disposal and fosters the creation of sustainable regional production chains.^16,17^

The present work seizes this opportunity for the first time by reporting the conversion of tucumã peel into KOH-activated porous carbon for application as high-performance electrodes in supercapacitors. The novelty of this study lies in demonstrating how an abundant and low-cost Amazonian biomass can be transformed into a functional carbon material with high specific surface area, hierarchical porosity, and excellent electrochemical performance. This approach underscores the importance of integrating technological development with the regional bioeconomy, creating a model for valorizing Amazonian residues that combines economic, social, and environmental benefits and contributes to the advancement of sustainable materials for next-generation energy-storage devices.

## 2 Materials and Methods

### 2.1 Materials

Tucumã (*A. aculeatum*) peel residues were collected in Manaus, Amazonas, Brazil. The material was ground using a ball mill (EDG Equipamentos, Brazil) and subsequently dried at 100 °C for 72 h in a laboratory oven (Quimis model Q318M). For hydrothermal carbonization, a 50 mL Teflon vessel encased in a stainless-steel autoclave (Parr Instrument) was used. Potassium hydroxide (KOH, purity ≥ 85%, Dinâmica Química Contemporânea, Brazil) was employed for the chemical activation of the biochar/KOH mixtures using a horizontal tubular furnace (FT20 HI-220 V, EDG, Brazil) equipped with a 50 mm diameter quartz tube (99.99% SiO_2_, Heraeus Quartz, Germany).

The samples were placed in alumina crucibles (≥99% Al_2_O_3_, Alfa Aesar, USA) and subjected to thermal treatment under a nitrogen atmosphere (N_2_, purity 99.99%, White Martins, Brazil). A 1 mol L^–1^ hydrochloric acid solution was prepared by diluting concentrated HCl (37%, Synth, Brazil) in distilled water for washing the pyrolyzed product. For electrode ink preparation, isopropyl alcohol (Dinâmica, 99.8%), Vulcan carbon black (Cabot, 99.5%), and Nafion (Ion Power, 5.0%) were used.

The dispersions were homogenized via ultrasonic bath (USC-1800A, 40 kHz, 135 W, Unique, Brazil). The prepared ink was then applied onto 304 stainless steel substrates (0.5 × 1.0 cm). Electrochemical measurements were performed using an automatic potentiostat/galvanostat (PGSTAT 302 N, Metrohm Autolab).

### 2.2 Hydrothermal Carbonization of Tucumã Peel

The raw tucumã peel powder was initially subjected to hydrothermal carbonization, following a procedure adapted from Teixeira et al.^18^ to obtain the hydrochar precursor. Approximately 4 g of the dried and milled peel were dispersed in deionized water to form a homogeneous slurry and then transferred to a Teflon-lined stainless-steel autoclave. The sealed reactor was heated to 180 °C and maintained at this temperature for 24 h. Under these conditions, the lignocellulosic structure undergoes dehydration, polymerization, and aromatization reactions, yielding an oxygen-rich hydrochar with enhanced carbon content and an initial porous framework. After natural cooling to room temperature, the solid product was recovered by filtration, thoroughly washed with deionized water to remove soluble byproducts, and subsequently dried at 105 °C for 12 h. From the 4 g of initial biomass, 3.4 g of dry hydrochar were obtained, corresponding to a production yield of approximately 85%. This hydrochar was then used as the carbonaceous precursor for the subsequent chemical activation with KOH.

### 2.3 Synthesis of Activated Carbon from Tucumã Bark

Following the methodology described by Díaz et al.,^11^ porosity was induced through chemical activation by adding a 50 wt % aqueous KOH solution to 1 g of biochar, establishing biochar: KOH mass ratios of 1:1, 1:3, and 1:5. The resulting mixture was placed in alumina crucibles and thermally treated at 850 °C. The thermal profile involved a pretreatment at 130 °C for 12 h, followed by pyrolysis at 850 °C for 1 h under a nitrogen flow of 100 mL min^–1^ with a heating rate of 10 °C min^–1^ throughout all stages. After activation, the solids were washed with 1.0 M HCl and rinsed with deionized water, then dried at 100 °C for 12 h. The final samples were labeled as TAKx, where TAK denotes “tucumã activated with KOH” and x corresponds to the KOH ratios of 1, 3, and 5, respectively.

### 2.4 Physicochemical Characterization

The morphology of the samples was investigated via scanning electron microscopy (SEM; TESCAN VEGA3) at an accelerating voltage of 15.0 kV. Structural characterization was conducted by X-ray diffraction (XRD) over a 2θ range of 10–80° using a PANalytical Empyrean diffractometer. Raman spectroscopy was performed with a 488 nm He–Ne laser (HR800 Evolution Raman, HORIBA Scientific, Jobin-Yvon). Surface area and porosity analysis were conducted by nitrogen adsorption–desorption at 77 K using a Quantachrome NOVA 1000e instrument. Prior to analysis, the samples were degassed under vacuum at 200 °C for 2 h. The specific surface area was determined by the Brunauer–Emmett–Teller (BET) method over a relative pressure range of 0.05–0.8. Pore size distribution was obtained from nitrogen adsorption isotherms using density functional theory (DFT), based on a cylindrical pore model and the adsorption branch of the isotherms, allowing for precise quantification of micro- and mesoporous fractions.

### 2.5 Electrode Fabrication

The working electrode was prepared by dispersing a homogeneous mixture containing 85 wt % activated carbon, 10 wt % Vulcan carbon as a conductive agent, and 5 wt % Nafion as a binder in isopropyl alcohol, forming a uniform suspension. The suspension was brush-coated onto a stainless-steel substrate. Approximately 1.0 mg of active material, measured with an analytical balance of ±0.01 mg precision, was deposited and uniformly distributed across three electrodes to ensure consistent coating and strong adhesion to the substrate. After complete drying, the resulting film exhibited an average thickness of approximately 210 μm.

### 2.6 Electrochemical Measurements

Electrochemical tests were conducted using a PGSTAT 302 N potentiostat/galvanostat (Metrohm Autolab) in a three-electrode configuration, employing a saturated Ag/AgCl reference electrode, a platinum foil as counter electrode, and the porous carbon-coated steel as the working electrode in 1 M KOH electrolyte. Cyclic voltammetry, galvanostatic charge–discharge (GCD), and electrochemical impedance spectroscopy (EIS) were employed. CVs were performed in the potential window of −1.0 to 0.0 V at scan rates ranging from 5 to 200 mV s^–1^. GCD curves were obtained over the same voltage range at current densities from 1 to 9 A g^–1^. Electrochemical stability was evaluated at 7 A g^–1^ using GCD. EIS measurements were carried out over a frequency range of 10^4^ to 10^–2^ Hz with a perturbation amplitude of 10 mV. The specific capacitance (*C*_sp_) was calculated from GCD data using the following equation

1 $$C_{sp}=\frac{I\times \Delta t}{\Delta V\times m}$$

in eq 1, Δ*t*, Δ*V*, *I*, and *m* represent the discharge time, potential window, applied current density, and mass of the active material, respectively.

The electrochemically active surface area (ECSA) of the porous carbon electrodes was estimated from the specific capacitance (*C*_sp_) obtained in 1 M KOH, assuming a typical double-layer specific capacitance of carbon materials of 20 μF cm^–2^.^19^ The ECSA was calculated according to

2 $$ECSA\;\left({m^{2}g}^{-1}\right)=\frac{C_{sp}\;\left(F{g^{-}}^{1}\right)}{C_{dl}\;\left(Fm^{-2}\right)}$$

where *C*_dl_ is the typical double-layer capacitance (20 μF cm^–2^ ≈ 0.2 F m^–2^), this procedure allows direct comparison between the geometrical surface area determined by nitrogen adsorption (BET) and the electrochemically accessible area.

Using impedance measurements, one can also study the complex potential, which is considered useful in terms of system behavior from the electrical point of view. Capacitive materials vary between resistive states at high frequency and capacitive states at low frequency, and the power is expressed according to equations

3 $$C=-\frac{Z^{\prime \prime }\left(\omega \right)}{\omega \;{\left[Z\left(\omega \right)\right]}^{2}}$$

4 $$C^{\prime \prime }=-\frac{Z^{\prime }\left(\omega \right)}{\omega \;{\left[Z\left(\omega \right)\right]}^{2}}$$

5 S(ω)=P(ω)+jQ(ω)

6 $$P\left(\omega \right)=\frac{\omega C^{\prime \prime }\left(\omega \right)}{{\left|{\Delta V}_{rms}\right|}^{2}}$$

7 $$Q\left(\omega \right)=\frac{-\omega C^{\prime }\left(\omega \right)}{{\left|{\Delta V}_{rms}\right|}^{2}}$$

where *C*′(ω) is the real part of the complex capacitance and C″(ω) is the imaginary part of the complex capacitance that is expressed by *C* (ω), these being the *Z*″(ω) and *Z*″(ω) respectively, which are the real and imaginary regions of the complex impedance, while the ω is the angular frequency shown by ω = 2π*f*. The parameters *C*′(ω) and *C*″(ω) are the capacitances of the active electrode material and the energy dissipation respectively, which is expressed by the irreversibility of the process, which leads to a hysteresis of the material.

The power is composed of the real and imaginary parts, the active powers being *P*(ω) and the reactive powers being *Q*(ω), and |Δ*V*_rms_|^2^ = $\frac{\Delta V}{\sqrt{2}}$ , with Δ*V*_max_ being the maximum amplitude of the alternating current (AC) signal. Through the intercession of |*Q*| *S* | = /*P*| *S*/, which corresponds to the relaxation time constant, τ_0_ = $\frac{1}{2f\pi }$ , this parameter defines the boundary where the material passes from resistive to capacitive states or vice versa.

## 3 Results and Discussion

### 3.1 Influence of Potassium Hydroxide Activation on the Physicochemical Properties of Porous Carbons

Chemical activation using potassium hydroxide (KOH) is a well-established method for producing high-performance porous carbons. It is widely employed due to its effectiveness in generating large specific surface areas and a well-developed porous network. The activation process involves a combination of redox reactions, chemical etching, and gas evolution, which promote the formation of both micropores and mesopores within the carbon matrix.^20^

During the activation stage, performed at 850 °C for 1 h, solid KOH melts and reacts with the carbon in the biochar, leading to the formation of intermediate compounds such as potassium carbonate (K_2_CO_3_) and potassium oxides (K_2_O).^21^ These species interact with the carbonaceous structure through a series of reactions, which can be summarized as follows

8 $${6KOH+2C\to 2K+3H}_{2}{+2K}_{2}{CO}_{3}$$

9 $$K_{2}{CO}_{3}{\to K}_{2}{O+CO}_{2}$$

10 $${CO}_{2}+C\to 2CO$$

These reactions release gases such as H_2_, CO_2_, and CO, which generate internal pressure within the carbon matrix, promoting structural expansion and pore formation.^22^ Simultaneously, the metallic potassium formed can intercalate between graphitic layers, further enhancing the development of the porous network.^23^

As illustrated in Figure 1a–c, SEM images clearly demonstrate the effect of KOH activation on the morphology of the biochar. Well-defined and interconnected pores at multiple scales are observed, resulting from controlled chemical etching and gas evolution during activation. In image (a), slit-shaped pores of various sizes are uniformly distributed over the surface, indicating efficient and homogeneous chemical attack.

**Figure 1:**
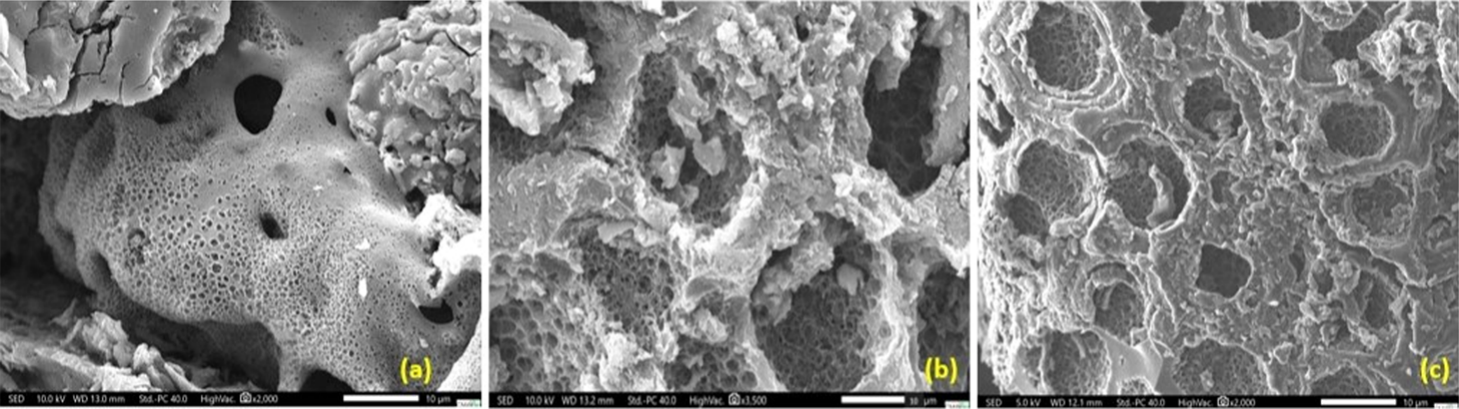
Scanning electron microscopy images of activated carbons treated at 850 °C with different KOH dosages: (a) TAK1, (b) TAK3, and (c) TAK5.

Image (b) reveals the formation of highly porous structures, with thin walls and voids attributed to the removal of amorphous carbon phases. In image (c), circular-shaped pores with homogeneous distribution and finely textured walls are evident an outcome of the combined action of high KOH concentration and elevated activation temperature. Similar morphologies have been reported by other authors^24^ although they employed higher KOH dosages, which ultimately led to degradation of the carbon matrix.

The biochar-to-KOH ratio plays a crucial role in controlling the extent of chemical activation, directly influencing the porosity and surface area of the resulting materials.^19^ The specific surface area represents the amount of active surface available per unit mass of the material,^25^ while porosity-defined by the total pore volume and pore size distribution-directly affects the diffusion of ions and molecules within the material, making it a key feature for supercapacitor applications.^26^ ^27^

Higher KOH ratios (1:3 and 1:5) provide a greater amount of activating agent, leading to more intense chemical etching, removal of amorphous content, and additional pore formation, resulting in materials with larger specific surface areas and total pore volumes.^28^ Conversely, the 1:1 ratio produces porous carbons with a lower degree of activation, higher carbon yield, but reduced pore accessibility.

These trends were confirmed by the nitrogen adsorption–desorption isotherms shown in Figure 2a. The TAK1 sample exhibited type I­(a) isotherms, characteristic of predominantly microporous materials (<2 nm), whereas TAK3 and TAK5 displayed type I­(b) isotherms, indicating the presence of larger micropores and small mesopores (∼2.5 nm), according to IUPAC classification. Adsorption was primarily observed at relative pressures below 0.8 *P*/*P*_0_, highlighting the accessibility of micropores. The specific surface areas (SSA) for the samples were 636 m^2^ g^–1^ (TAK1), 1932 m^2^ g^–1^ (TAK3), and 2468 m^2^ g^–1^ (TAK5), confirming that increasing the KOH ratio significantly enhances pore development and surface availability for adsorption.

**Figure 2:**
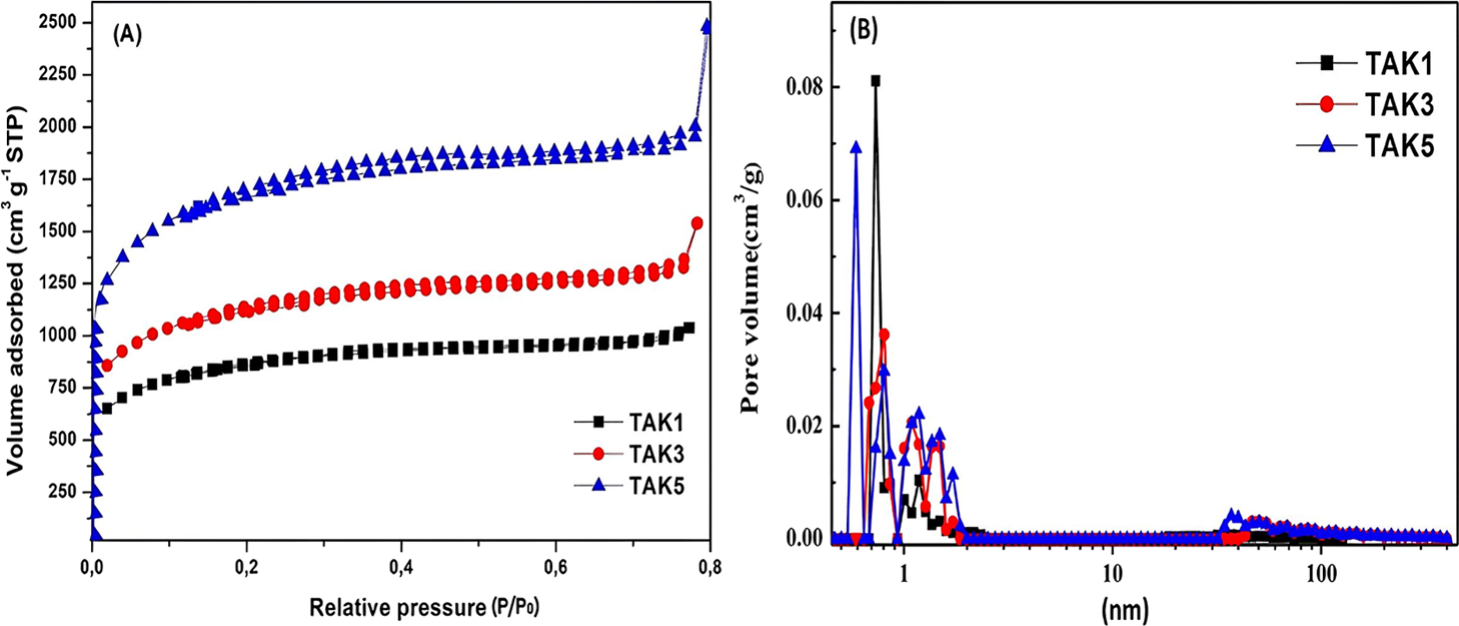
(A) Nitrogen adsorption–desorption isotherms of TAK1, TAK3, and TAK5; (B) corresponding pore size distributions calculated using the DFT method.

The pore size distribution analysis shown in Figure 2B reveals that samples TAK3 and TAK5 exhibit prominent peaks between 0.5 and 2 nm, characteristic of micropores, along with an additional peak around 2.5 nm, indicating the presence of mesopores. In contrast, sample TAK1 shows a predominant peak below 0.8 nm, suggesting a dominant microporous structure.

Recent studies^28,29^ demonstrate that pore hierarchy namely, the balanced combination of micropores, mesopores, and, to a lesser extent, macropores plays a decisive role in the performance of materials designed for electrochemical charge storage. In particular, the volume and distribution of micropores are crucial, as these cavities provide a large internal surface area that promotes the formation of the electric double layer and, consequently, enhances the specific capacitance through ion adsorption at the electrode/electrolyte interface.^30^

Mesopores, on the other hand, act as transport channels, allowing ions to rapidly diffuse into the deeper microporous regions. This mesoporous network reduces ionic diffusion resistance and increases the accessibility of active sites, which translates into improved charge–discharge response, especially at high current densities.^31^

When present in a well-balanced configuration, this micro/mesopore hierarchy combines the high charge-storage capacity provided by the micropores with the fast transport kinetics ensured by the mesopores, resulting in superior electrochemical performance, enhanced cycling stability, and more efficient utilization of the material in devices such as supercapacitors.

As shown in Table 1, increasing the KOH/biochar ratio consistently enhances the pore volumes of the samples. TAK1 exhibited a total pore volume of 0.28 cm^3^ g^–1^, predominantly composed of micropores (0.14 cm^3^ g^–1^), with a relatively low mesopore volume (0.03 cm^3^ g^–1^). In contrast, TAK3 showed a substantial increase in total pore volume to 1.32 cm^3^ g^–1^, with micropore and mesopore volumes of 0.23 cm^3^ g^–1^ and 0.21 cm^3^ g^–1^, respectively. For TAK5, the total pore volume reached 1.91 cm^3^ g^–1^, comprising 0.31 cm^3^ g^–1^ of micropores and 0.43 cm^3^ g^–1^ of mesopores.

**Table 1:** Specific Surface Area and Pore Structure of TAK1, TAK3, and TAK5 Samples Activated at 850 °C Using Different KOH Dosages

sample	*S*_APOSTA_ (m^2^ g^–1^) specific surface area	*V*_t_ (cm^3^ g^–1^) total pore volume	*V*_um_ (cm^3^ g^–1^) micropore volume	*V*_me_ (cm^3^ g^–1^) mesopore volume
TAK1	636	0.28	0.14	0.03
TAK3	1932	1.32	0.23	0.21
TAK5	2468	1.91	0.31	0.43

The observed trend demonstrates that activation with higher KOH ratios not only increases the number of micropores but also significantly promotes the development of mesopores, resulting in a hierarchical pore structure. This hierarchy is crucial for applications in supercapacitors.

In addition to the pore structure, the crystallinity and structural ordering of the carbon materials were investigated by X-ray diffraction (XRD), as shown in Figure 3a. All samples exhibited two characteristic diffraction peaks at approximately 2θ ≈ 26.4° and 43.5°, which are attributed to the (002) and (100) planes of graphitic carbon, respectively.^32^ For the TAK1 sample, the peaks appeared with lower intensity in the low-angle region, indicating a more disordered carbon structure. In contrast, TAK3 and TAK5 showed sharper and more intense peaks in the same region, suggesting a decrease in amorphous carbon content or an increase in the degree of structural order with higher KOH dosages.^33^

**Figure 3:**
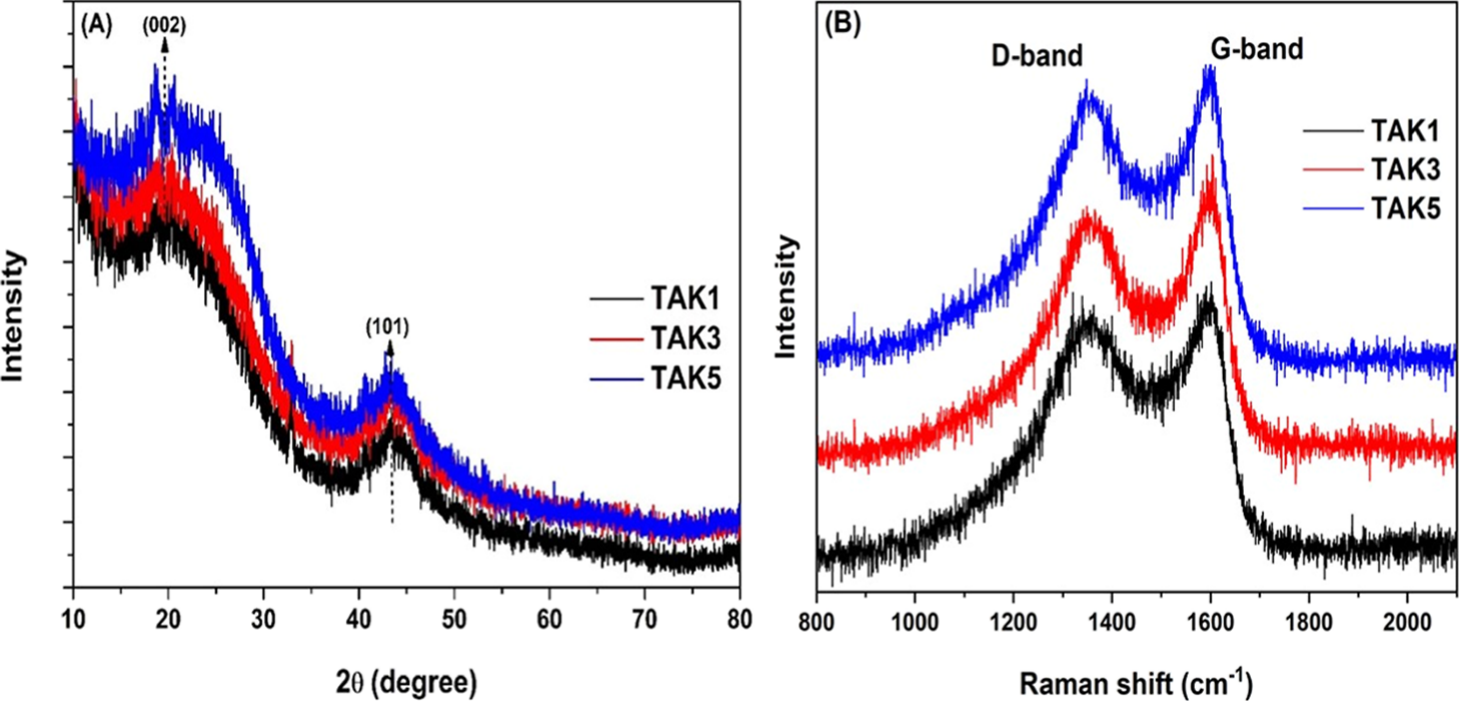
Structural characterization of activated carbons TAK1, TAK3, and TAK5. (A) X-ray diffraction (XRD) showing peaks corresponding to the (002) and (100) planes. (B) Raman spectra with D and G bands.

The progressive enhancement in peak intensity from TAK3 to TAK5 further supports the findings from the textural analysis, indicating that higher activation levels not only promote the development of hierarchical porosity but also enhance the structural organization of the carbon framework. This evolution suggests improved stacking of graphitic layers and the formation of more ordered domains at elevated KOH concentrations.

The XRD results are corroborated by Raman spectroscopy data presented in Figure 3b, which provide complementary information regarding the degree of graphitization and the presence of structural defects in the KOH-activated materials.

Raman spectroscopy was employed to investigate the structure of the activated carbon materials, enabling the assessment of disorder and the presence of graphitic domains. The spectra displayed two characteristic bands: the D band (∼1350 cm^–1^), associated with structural defects and edges of the graphitic layers (related to sp^3^ vibrational modes), and the G band (∼1580 cm^–1^), corresponding to the in-plane vibration of sp^2^-hybridized carbon atoms in ordered graphitic structures.^34,35^

The intensity ratio of the D and G bands (*I*_D_/*I*_G_) is a key parameter for estimating the degree of disorder and the defect density in the material; lower values indicate a lower level of disorder.^36^ For the analyzed samples, *I*_D_/*I*_G_ values were approximately 0.98 for TAK1, 0.95 for TAK3, and 0.92 for TAK5, indicating that increasing the KOH ratio during activation led to a slight reduction in structural disorder.

This gradual decrease in the *I*_D_/*I*_G_ ratio suggests that, although KOH activation promotes defect formation and porosity development, higher KOH dosages favor the removal of amorphous carbon and the exposure of slightly more ordered graphitic domains-an effect previously reported in the literature.^37^ ^,^ ^38^ It is also consistent with the notion that the growth of crystalline carbon and improved stacking of carbon layers occur more effectively at higher activation concentrations.

Together, these results confirm that the activated materials possess a disordered graphitic structure with partially ordered regions, maintaining a balance between porosity, active sites, and electrical conductivity-features that are highly desirable for energy storage applications.

### 3.2 Electrochemical Performance of Porous Carbons Derived from Tucumã Peels Activated with KOH

Cyclic voltammetry is an electrochemical technique commonly used to evaluate the capacitive behavior of electrode materials, enabling the analysis of current response as a function of the applied potential in a three-electrode system. For carbon-based materials, rectangular or quasi-rectangular CV profiles are indicative of capacitive behavior governed by electric double-layer charge storage (EDLC),^39^ whereas distortions or redox peaks may suggest pseudocapacitive contributions due to the presence of surface functional groups or heteroatoms.^40^

Figure 4 presents the cyclic voltammograms of TAK1, TAK3, and TAK5 recorded at a scan rate of 50 mV s^–1^ in 1 M KOH aqueous solution. All samples exhibit quasi-rectangular profiles across the entire potential window (−1.0 to 0.0 V vs Ag/AgCl), characteristic of EDLC behavior, confirming that the porous carbons derived from tucumã peels function as efficient capacitive materials.

**Figure 4:**
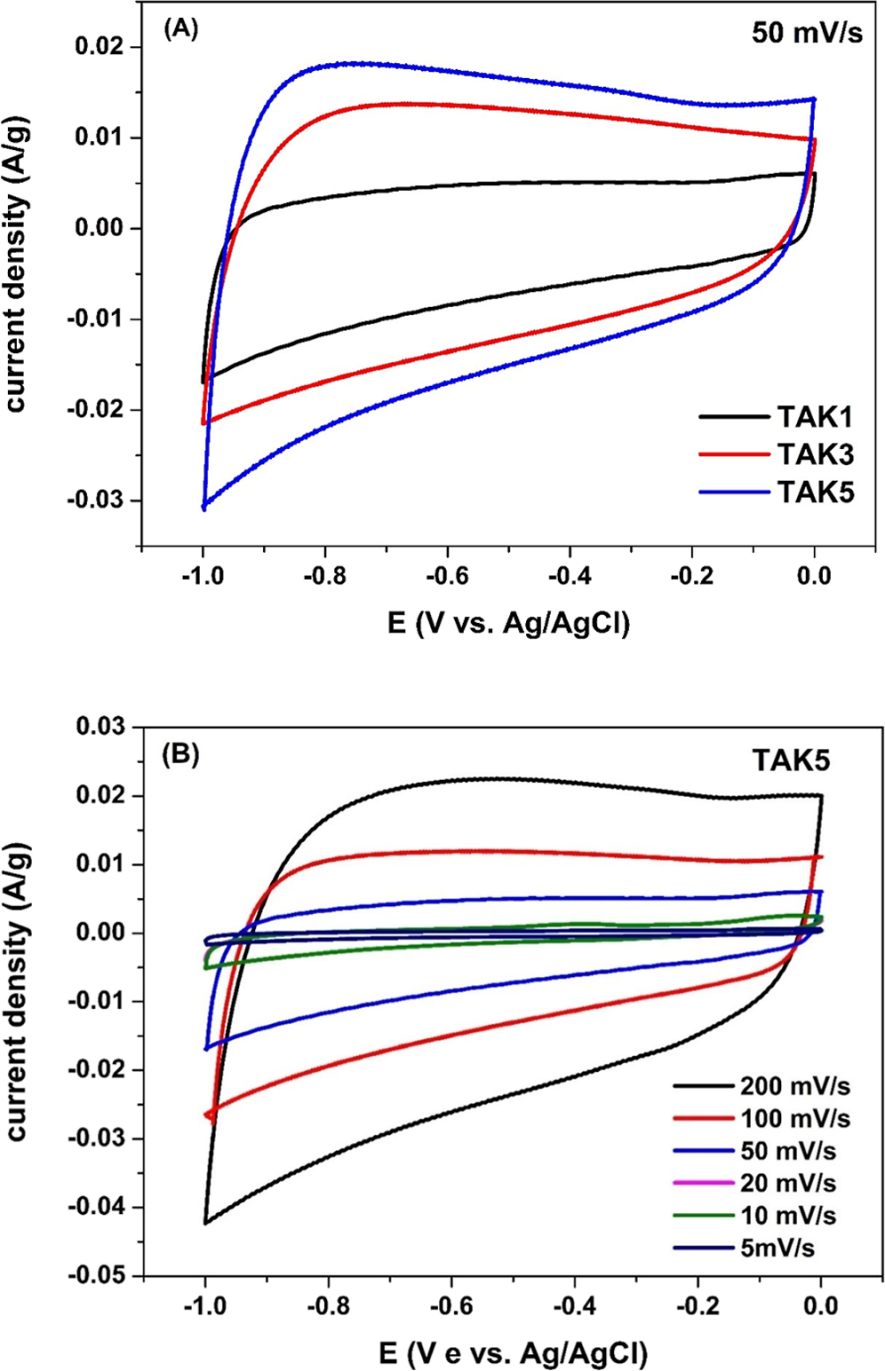
(A) Cyclic voltammograms of TAK1, TAK3, and TAK5 electrodes recorded at a scan rate of 50 mV s^–1^ in the potential range of −1.0 to 0.0 V. (B) Cyclic voltammograms of the TAK5 electrode at various scan rates.

Among the samples, TAK5 displayed the largest area under the CV curve, followed by TAK3 and TAK1, indicating that increasing the KOH ratio during activation is directly associated with enhanced specific capacitance.

When compared with other electrode materials reported in recent studies such as the CrB MBenes of Aghamohammadi et al.,^41^ the La_1–*x*_Ce_*x*_CoO_3_–δ perovskites of Cao et al.,^42^ and the SrCo_0.925_Sc_0.075_O_3_–δ electrodes of He et al.^43^ it is possible to establish relevant parallels with the behavior of the TAK5 material.

The CrB MBenes, synthesized from the MAB phase Cr_2_AlB_2_ by selective etching in 1.25 M HCl, exhibited a two-dimensional lamellar morphology and a specific capacitance of 92.3 F g^–1^ at 0.1 A g^–1^, with a strong pseudocapacitive contribution (86.46%). This high participation of faradaic processes contrasts with TAK5, whose charge storage occurs mainly through electric double-layer formation. This difference highlights distinct energy-storage mechanisms: while CrB MBene relies on surface redox reactions, TAK5 is governed by electrostatic adsorption within its porous network.

The La_1–*x*_Ce_*x*_CoO_3_–δ perovskites, prepared via electrospinning-calcination, reached 267.9 F g^–1^ at 1 A g^–1^ when *x* = 0.1, attributed to the increase in oxygen vacancies and the stabilization of the cubic phase. In comparison, TAK5 operates within a wider potential window, whereas the perovskites despite their higher redox activity typically exhibit a narrower range of about 0.6 V. This difference reflects the greater pseudocapacitive contribution of the perovskites compared to the predominantly electric double-layer behavior of the biochar.

The SrCo_0.925_Sc_0.075_O_3_–δ electrode developed by Ele et al. exhibited a capacity of 467.7 C g^–1^ (≈129.9 mAh g^–1^) at 1 A g^–1^, with 97.4% retention after 10,000 cycles, benefiting from a 3D dual-network gel design that enhanced both stability and flexibility. Although this electrode-engineering strategy differs markedly from the biochar approach, both materials demonstrate good cycling stability, albeit achieved through different mechanisms TAK5 through the robustness of its porous carbon framework and Ele’s material through the mechanical support of the conductive gel.

These comparisons underscore how different synthesis route-from 2D MBene structures to perovskites with engineered oxygen vacancies and conductive gel networks, result in unique combinations of capacitance, stability, and charge-storage mechanisms. Within this context, TAK5 exhibits a profile dominated by electric double-layer capacitance, demonstrating that biomass-derived carbons can compete in performance while exploiting electrochemical principles distinct from those of the oxide-based and 2D materials reported in the literature.

Figure 4B displays the cyclic voltammograms of TAK5 obtained at different scan rates ranging from 5 to 200 mV s^–1^. The CV profiles maintain a quasi-rectangular shape across all scan rates, indicative of capacitive behavior governed by electric double-layer capacitance (EDLC), even at high scan rates.^20,44^ This behavior reflects the good reversibility of the charge/discharge processes and the fast capacitive response of the material.

As the scan rate increases, a progressive rise in current density is observed, demonstrating the material’s ability to deliver higher currents under fast charging conditions. The slight distortion of the curves at high scan rates, such as at 200 mV s^–1^, is attributed to internal resistance and diffusion limitations, phenomena commonly reported for porous carbon materials.^45,46^

This electrochemical behavior is closely linked to the structural properties discussed in Section 3.1, such as high specific surface area and large total pore volume. Furthermore, the moderate structural disorder revealed by Raman analysis and the presence of partially ordered graphitic domains (as evidenced by the (002) XRD peak) contribute to adequate electrical conductivity, facilitating efficient charge transport within the electrode.^47,48^

Figure 5 presents the galvanostatic charge–discharge (GCD) curves of samples TAK1, TAK3, and TAK5 at a current density of 1 A g^–1^. All samples exhibit symmetric triangular profiles, characteristic of capacitive behavior dominated by EDLC mechanisms, indicating good electrochemical reversibility and low internal resistance.^49,50^ Among the samples, TAK5 exhibited the longest discharge time, followed by TAK3 and TAK1. This progressive increase in discharge time is directly associated with enhanced specific capacitance (*C*_sp_), with values of 84, 146, and 298 F g^–1^, respectively, calculated using eq 1, indicating that TAK5 has the highest charge storage capability.

**Figure 5:**
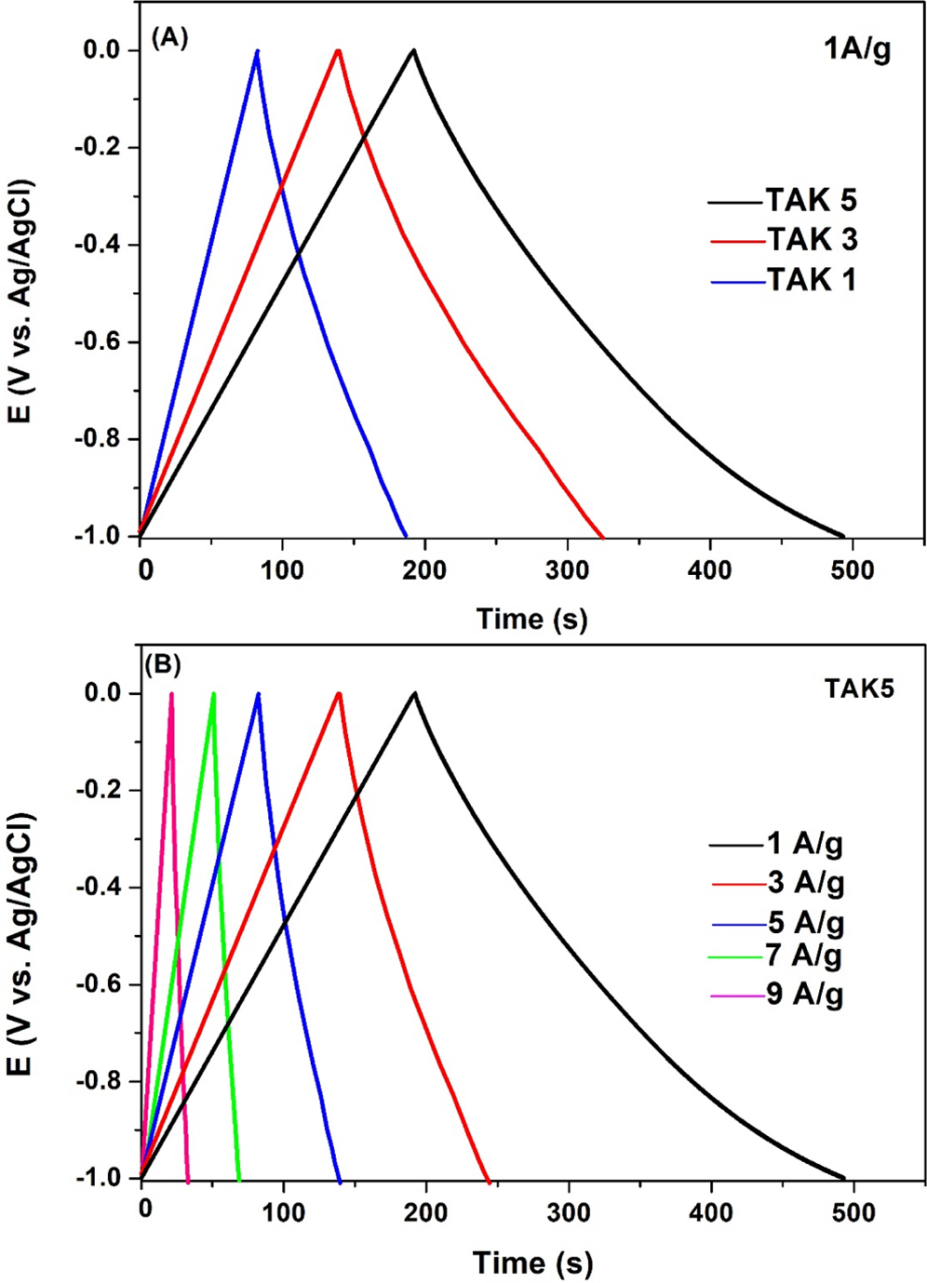
(A) Galvanostatic charge–discharge curves in the potential range from −1.0 to 0.0 V for TAK1, TAK3, and TAK5 electrodes. (B) TAK5 curves at different current densities.

This improved performance can be attributed to the activation process using higher KOH ratios, which resulted in greater specific surface area (SSA) and total pore volume for TAK5 (2468 m^2^ g^–1^ and 1.91 cm^3^ g^–1^, respectively), as previously discussed. The high SSA and hierarchical porosity favor efficient ion diffusion throughout the carbon matrix, enabling greater ion accessibility to active sites during charge–discharge cycles, thereby enhancing capacitance.^27^

To deepen the relationship between the increase in specific surface area (SSA) and the improvement in specific capacitance (*C*_sp_), the electrochemically active surface area (ECSA) was estimated from the specific capacitance obtained in 1 M KOH according to eq 2, considering a typical double-layer capacitance for carbon materials of about 20 μF cm^–2^.^51^ Based on the gravimetric capacitance of 298 F g^–1^ for the TAK5 electrode, the calculated ECSA is approximately 1.5 × 10^3^ m^2^ g^–1^, a value almost six times higher than the BET surface area (≈2468 m^2^ g^–1^) determined from N_2_ adsorption.

This difference, commonly reported for highly porous carbons, indicates that the effective charge-storage interface extends beyond the geometric area measured by BET and is generally attributed to the presence of micropores and internal surfaces that become electrochemically accessible in the electrolyte, allowing the formation of an electrical double layer much larger than that predicted solely from gas-adsorption data.^30^

Moreover, the linear and symmetric shape of the GCD curves suggests efficient charge storage behavior with no significant faradaic processes, confirming the predominance of the EDLC mechanism in this system. Similar results have been reported by other authors^52,53^ using KOH-activated carbon materials, also demonstrating that higher surface areas and pore volumes are correlated with longer discharge times and higher specific capacitance in supercapacitor applications.

Therefore, the GCD results are in strong agreement with the cyclic voltammetry data presented earlier, further reinforcing that the progressive increase in the KOH: biomass ratio during activation is directly related to enhanced electrochemical performance.

Figure 5 displays the galvanostatic charge–discharge (GCD) profiles of the TAK5 sample at various current densities (1, 3, 5, 7, and 9 A g^–1^). All curves exhibit a symmetrical triangular shape, typical of electric double-layer capacitors (EDLCs), even at high current densities. This behavior indicates a predominantly capacitive charge storage mechanism. As the current density increases, a progressive decrease in the discharge time is observed, which is expected due to reduced ion diffusion within the porous structure at higher charge/discharge rates.^49,54^

The electrochemical performance of the TAK5 sample can be compared with the results summarized in Table 2. The specific capacitance achieved by TAK5 (298 F g^–1^ in 1 M KOH) stands out compared to other biomass-derived carbons, highlighting the superior capacitive performance of this material. This enhancement can be attributed to the synthesis conditions employed (hydrothermal carbonization followed by KOH-assisted pyrolysis), as well as to the favorable porous structure, which promotes efficient ion adsorption/desorption even under high current densities.

**Table 2:** Carbon Electrodes Synthesized from Different Biomass Precursors and Methods, Showing a Range of Specific Capacitances

biomass	summary/agent activator	and electrolyte	capacitance specific F/g	reference
straw waste	pyrolysis/ZnO	6 M KOH	220.7	^55^
poplar sawdust	carbonization hydrothermal/pyrolysis of H_3_PO_4_	6 M KOH	161	^56^
tamarind seed husk	pyrolysis/KOH	6 M KOH	157	^57^
shell Ricinus communis	carbonization hydrothermal/KOH pyrolysis	3 M KOH	137	^53^
carbon Garlic Seeds	pyrolysis/KOH	6 M KOH	268	^52^
baobab fruit peels	pyrolysis/H_3_PO_4_	6 M KOH	233	^58^
this work	carbonization hydrothermal/KOH pyrolysis	1 M KOH	298	this work

Continuing the electrochemical evaluation, Figure 6A shows the variation in specific capacitance of the TAK5 sample at different current densities (1, 3, 5, and 7 A g^–1^). It is observed that as the current density increases, the specific capacitance gradually decreases from approximately 298 F g^–1^ at 1 A g^–1^ to about 160 F g^–1^ at 7 A g^–1^.

**Figure 6:**
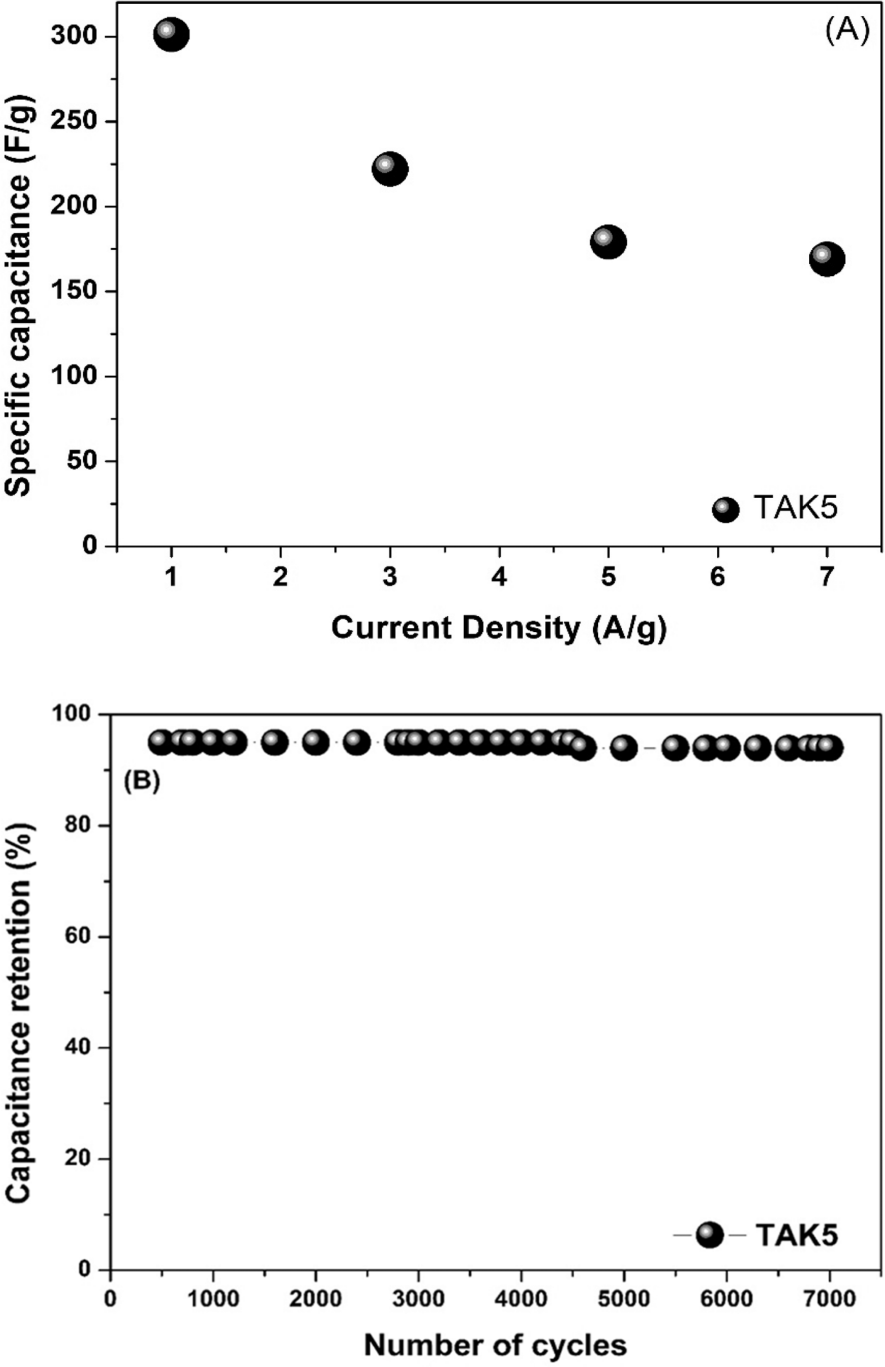
(A) Analysis of the variation in current density vs specific capacitance; (B) cycling performance of TAK5.

This behavior is characteristic of porous materials used in supercapacitors, as at high current densities, ion diffusion in the electrolyte becomes limited, reducing the utilization of the active surface area of the electrode, as previously mentioned. Despite this reduction, the TAK5 sample maintains high specific capacitance values even under severe operating conditions, indicating good electrical conductivity and accessible porous architecture.

Similar behavior is reported in previous studies.^59,60^ However, in certain materials, increasing the current density does not result in a satisfactory electrochemical response, even making it impossible to calculate specific capacitance. This behavior may be attributed to the low electrical conductivity of the material or to a poorly accessible porous structure^61^ that hinders efficient ion diffusion at high charge/discharge rates. An example of this limitation is reported by Manimekala et al. (2023),^62^ where only very low current densities (0.5 and 1 A g^–1^) were applied, likely due to the inability of the material to maintain capacitive performance under more demanding conditions.

Figure 6B illustrates the cycling stability of the TAK5 sample over 7000 charge–discharge cycles. A high capacitance retention, close to 100%, is observed, indicating excellent electrochemical stability and resistance to structural degradation over time. This performance highlights the robustness of the material as well as its compatibility with the electrolyte, making it a promising candidate for long-term energy storage applications such as supercapacitors.

The electrochemical impedance spectroscopy (EIS) analysis for the TAK5 material is presented in Figure 7A. The experimental Nyquist plot was fitted (*R* ^2^ = 0.99987) using the complex nonlinear least squares (CNLS) method with the equivalent circuit *R*_s_–(CPE || Rct),^61^ as illustrated in the upper corner of the figure. The solution resistance (*R*_s_ ≈ 1.75 Ω) indicates good ionic conduction within the electrolyte, suggesting that it effectively penetrates the active sites of the activated carbon, facilitating rapid ion adsorption and desorption.

**Figure 7:**
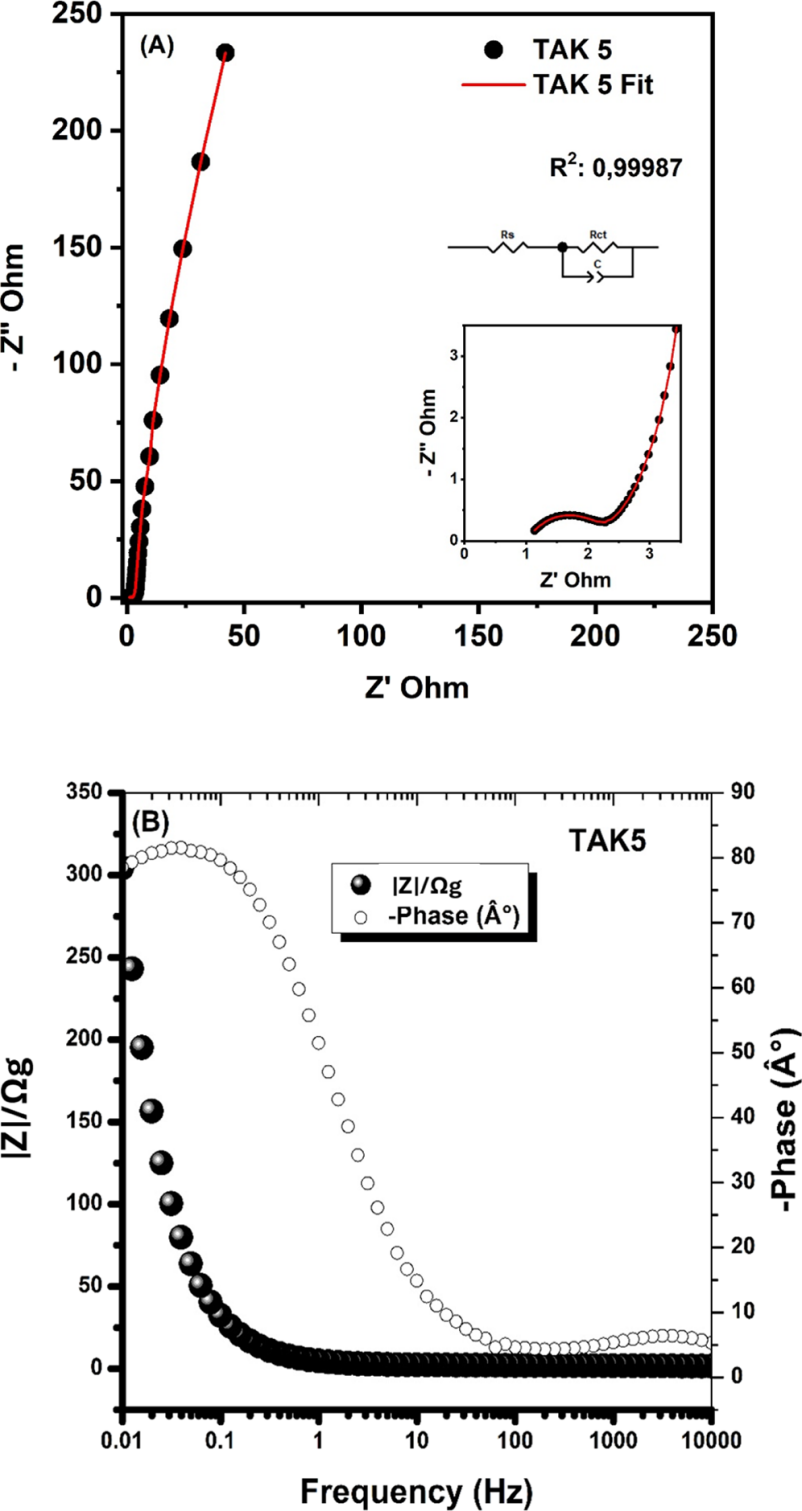
(A) Nyquist and (B) Bode plots of the TAK5 electrode obtained from electrochemical impedance spectroscopy (EIS), normalized by the active mass (0.1 mg). The data were fitted using the CNLS method with the equivalent circuit *R*_s_–(CPE || Rct).

Moreover, the charge transfer resistance (*R*_ct_ ≈ 0.25 Ω) is relatively low, reflecting fast electron/ion transport through the conductive porous framework of TAK5. The inclusion of the CPE (φ < 1) instead of an ideal capacitor is justified by the heterogeneous and porous nature of the electrode surface, which leads to phase dispersion typically observed in activated carbon-based electrodes.

Figure 7B presents the Bode plot, a fundamental representation for analyzing dynamic systems, particularly regarding frequency response. The impedance magnitude is represented by *Z*, and the diagram illustrates how this magnitude varies as a function of the frequency applied to the system. It is observed that, at high frequencies, the magnitude of *Z* is relatively low. This indicates that, under such conditions, the material or system offers little resistance to the flow of electric current.^63^

The impedance at high frequencies may be associated with predominantly capacitive behavior, where the current can flow more easily and the (Rct) is minimal, as also seen in the Nyquist plot. As the frequency decreases and approaches values below 1 Hz, the magnitude of Z begins to increase significantly. This increase suggests that the material or system starts to exhibit greater resistance to current flow, which may be related to resistive or inductive phenomena that become more prominent at low frequencies.^64^ In electrochemical systems, for instance, this may reflect contributions from slow diffusion processes or the intrinsic resistance of the material.^65^

Complementing the EIS analysis, the dielectric relaxation time constant (τ_0_), which reflects the dynamic response of the electrochemical system to frequency variation,^66^ was determined from the analysis of complex capacitance and complex power. This parameter is crucial for understanding the resistive and capacitive behavior of the electrode, allowing an assessment of its efficiency in fast charge–discharge processes, as required in high-power devices.

Figure 8A presents the variation of the imaginary part of the complex capacitance (C″) as a function of frequency. A well-defined peak is observed around 4 Hz, corresponding to a relaxation time τ_0_ ≈ 0.25 s, calculated using the equation τ_0_ = 1/*f*_max_, where *f*_max_ is the frequency at which (C″) reaches its maximum value. This result indicates that, for frequencies higher than f_0_, the system behaves predominantly as a resistor, whereas at lower frequencies, capacitive behavior becomes dominant.^67^ The presence of a low τ_0_ evidence that the TAK5 electrode has a high capacity for fast response, favoring efficient energy delivery.

**Figure 8:**
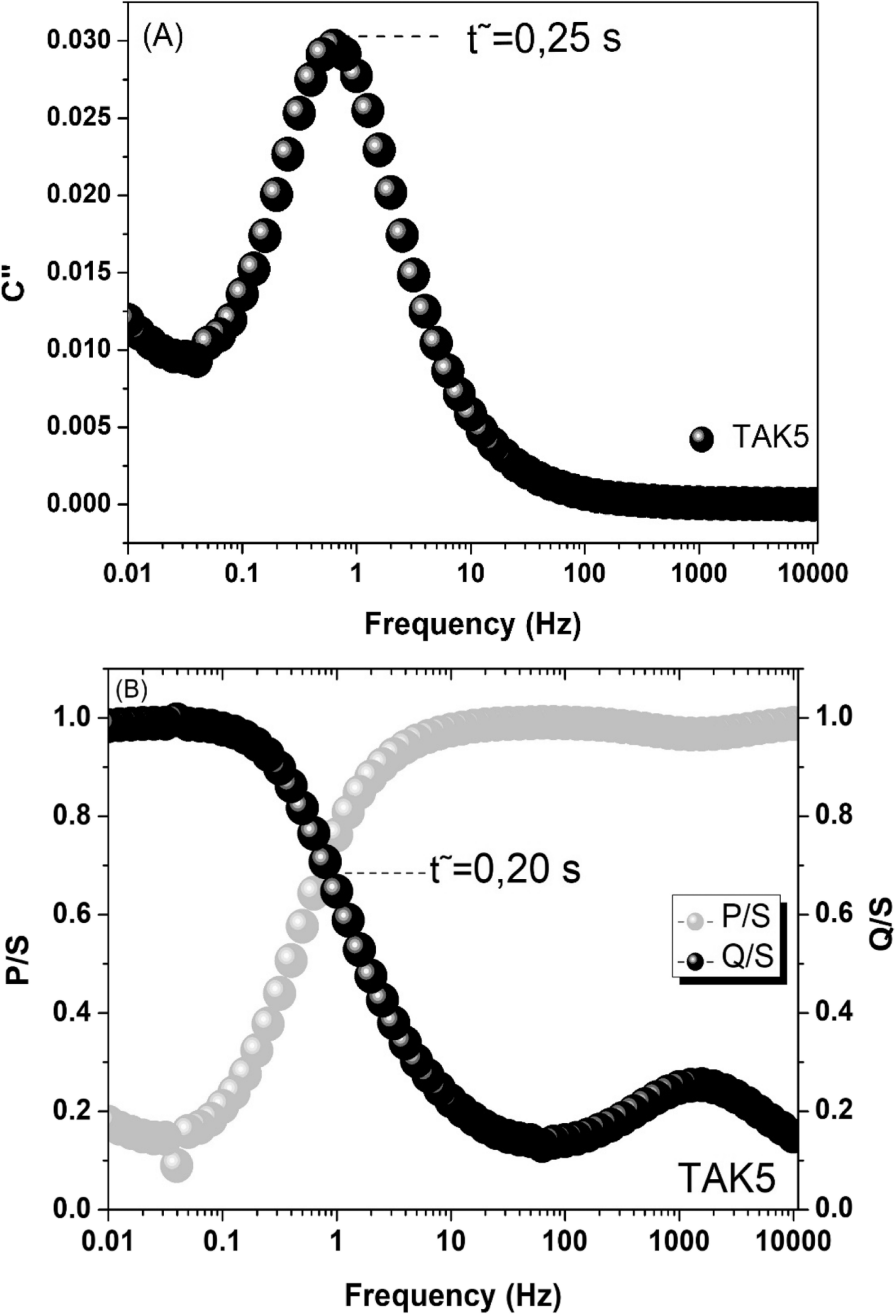
(A) Imaginary capacitance (*C*″) as a function of frequency for relaxation time (τ_0_) estimation of the TAK5 sample. (B) Normalized active and reactive power curves |*P*|/|*S*| and |*Q*|/|*S*|.

Additionally, Figure 8B shows the normalized active and reactive power curves, |*P*|/|*S*| and |*Q*|/|*S*|, respectively, as a function of frequency. At high frequencies, the materials behave like resistors and their powers are dissipated, presenting a high active power. While at low frequencies, the electrodes behave like capacitors and the reactive powers are high. The crossover point between these two curves occurs at approximately 0.20 s, which is in good agreement with the value obtained from the (C″) analysis. This point represents the optimal operating condition (0.7 in |*P*|/|*S*| and |*Q*|/|*S*|), where the energy stored and the power delivered by the electrodes are transferred with maximum efficiency.^66,68^

The consistency between the two methods used to determine τ_0_ reinforces the reliability of the data and demonstrates that the TAK5 electrode exhibits a highly responsive electrochemical profile. These results, combined with the previously observed low charge transfer resistance (*R*_ct_) and high cycling stability, indicate that the structure of TAK5 favors both efficient ion transport and rapid formation of the electrical double layer.

To better understand the mechanisms governing charge storage in the analyzed electrode, the method proposed by Dunn and Newman^69^ was applied. This is a widely used approach to separate and quantify the surface capacitive (EDLC) and diffusion-controlled pseudocapacitive (PC) contributions from cyclic voltammetry data collected at scan rates ranging from 5 to 100 mV s^–1^. This methodology is particularly useful for materials exhibiting hybrid electrochemical behavior, in which both surface adsorption and pseudocapacitive processes involving species diffusion occur simultaneously.^70^ The method is based on the dependence of current (*i*) on scan rate (*v*), according to the following equation

11 $${i\left(V\right)=k}_{1}{v+k}_{2}v^{1/2}$$

in this equation, the term *k*_1_ *v* represents the capacitive contribution, while the term *k*_2_ *v* ^1/2^ is related to the diffusion-controlled contribution, generally attributed to redox processes governed by ion transport within the electrode. For graphical analysis purposes, the equation is rearranged as follows

12 $$\frac{i\left(V\right)}{v^{1/2}}=k_{1}v^{1/2}{+k}_{2}$$

Based on this linearization, by plotting *i*/*v* ^1/2^ as a function of *v* ^1/2^, the slope of the resulting line corresponds to *k*_1_ (capacitive component), while the intercept represents *k*_2_ (diffusion-controlled component). With these parameters, it is possible to calculate the relative contributions of each mechanism at different scan rates. The application of Dunn’s method (Figure 9) revealed that the EDLC contribution prevails at all scan rates analyzed (5, 10, 25, 50, and 100 mV s^–1^), with increasing dominance as the scan rate increases ranging from 66.20% at 5 mV s^–1^ to 84.13% at 100 mV s^–1^.

**Figure 9:**
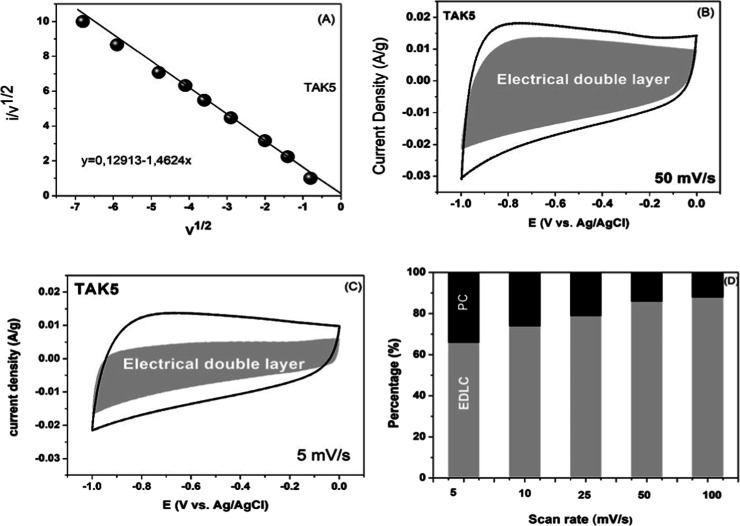
Analysis of the capacitive contributions of the TAK5 material using Dunn’s method. (A) Linear plot of *i*/*v* ^1/2^ vs *v* ^1/2^, indicating diffusion and electric double-layer capacitance (EDLC) contributions. (B–C) cyclic voltammetry curves at 50 and 5 mV s^–1^, respectively, showing the separation of regions corresponding to EDLC. (D) Percentage quantification of EDLC and pseudocapacitance (PC) contributions at different scan rates.

This behavior demonstrates that the material predominantly exhibits surface storage characteristics, typical of an electrode with dominant capacitive behavior.^70^ The small diffusion-controlled contribution, especially at low scan rates, may be associated with pseudocapacitance, confirming the presence of active functional groups on the material’s surface.^71^

## 4 Conclusion

In this study, tucumã bark was converted into activated carbon with interconnected porosity, encompassing micropores and mesopores, through hydrothermal carbonization followed by activation with KOH via pyrolysis. The resulting material was applied to the preparation of porous carbon electrodes for energy storage, with samples designated as TAK1, TAK3, and TAK5. Scanning electron microscopy (SEM) analyses revealed porosities, while nitrogen adsorption–desorption isotherms confirmed the presence of interconnected pores, with isotherms of types (a) and (b). The specific surface areas were 636 m^2^ g^–1^, 1932 m^2^ g^–1^, and 2468 m^2^ g^–1^ for TAK1, TAK3, and TAK5, respectively. X-ray diffraction (XRD) exhibited characteristic peaks at 24° and 43°, associated with the (002) and (101) crystal planes.

Raman spectroscopy revealed D and G bands, confirming the amorphous nature of the materials. Electrochemical results demonstrated nearly rectangular cyclic voltammetry curves for all samples, with the largest integrated area for TAK5. Galvanostatic charge–discharge (GCD) indicated high reversibility, with the formation of symmetrical triangles in the charge and discharge curves, and a longer discharge time for TAK5. The specific capacitances for TAK1, TAK3, and TAK5 were 84 F g^–1^, 146 F g^–1^, and 298 F g^–1^, respectively.

These results highlight that the carbon to KOH ratio (1:5) in TAK5 was crucial for the superior electrochemical performance, with higher specific surface area and capacitance. Sample TAK5 showed excellent electrochemical stability for up to 7000 cycles, along with low internal resistance (*R*_s_) and charge transfer resistance (*R*_ct_) values in electrochemical impedance spectroscopy analyses. Thus, this study demonstrates that the use of Amazonian wastes, such as tucumã (*A. aculeatum*) are excellent precursors for the synthesis of new materials capable of promoting sustainability and the bioeconomy. Furthermore, the limited research on the conversion of Amazonian biomass into energy storage devices represents an innovative area of study to be explored.
